# Improving Identification of Drug-Target Binding Sites Based on Structures of Targets Using Residual Graph Transformer Network

**DOI:** 10.3390/biom15020221

**Published:** 2025-02-03

**Authors:** Shuang-Qing Lv, Xin Zeng, Guang-Peng Su, Wen-Feng Du, Yi Li, Meng-Liang Wen

**Affiliations:** 1 Faculty of Surveying and Information Engineering, West Yunnan University of Applied Sciences, Dali 671000, China; 1220034@wyuas.edu.cn; 2 College of Mathematics and Computer Science, Dali University, Dali 671003, China; xinzeng@dali.edu.cn (X.Z.);; 3 State Key Laboratory for Conservation and Utilization of Bio-Resources in Yunnan, Yunnan University, Kunming 650000, China

**Keywords:** drug-target binding sites, structure, residual graph transformer network, residual 1D-CNN

## Abstract

Improving identification of drug-target binding sites can significantly aid in drug screening and design, thereby accelerating the drug development process. However, due to challenges such as insufficient fusion of multimodal information from targets and imbalanced datasets, enhancing the performance of drug-target binding sites prediction models remains exceptionally difficult. Leveraging structures of targets, we proposed a novel deep learning framework, RGTsite, which employed a Residual Graph Transformer Network to improve the identification of drug-target binding sites. First, a residual 1D convolutional neural network (1D-CNN) and the pre-trained model ProtT5 were employed to extract the local and global sequence features from the target, respectively. These features were then combined with the physicochemical properties of amino acid residues to serve as the vertex features in graph. Next, the edge features were incorporated, and the residual graph transformer network (GTN) was applied to extract the more comprehensive vertex features. Finally, a fully connected network was used to classify whether the vertex was a binding site. Experimental results showed that RGTsite outperformed the existing state-of-the-art methods in key evaluation metrics, such as F1-score (F1) and Matthews Correlation Coefficient (MCC), across multiple benchmark datasets. Additionally, we conducted interpretability analysis for RGTsite through the real-world cases, and the results confirmed that RGTsite can effectively identify drug-target binding sites in practical applications.

## 1. Introduction

Identification of drug-target binding sites (also known as pockets) is of great significance in drug screening and design [1,2]. For example, ATP (Adenosine triphosphate) binding sites play a crucial role in cellular-energy metabolism, signal transduction, and protein function. Over the years, the computational methods for identifying drug-target binding sites have evolved and can be broadly divided into two categories: traditional methods [3] and machine learning approaches [4,5,6,7]. Traditional methods include an energy-based approach Q-SiteFinder [8], which uses the interaction energy between the protein and a simple van der Waals probe to identify energetically favorable binding sites, a docking-based approach [9], which utilizes docking tools to locate the binding sites, and Fpocket, which is based on Voronoi tessellation and alpha spheres [10,11]. While some of these methods, such as Fpocket, have demonstrated strong performance in the identification of binding sites and are widely used, traditional methods are increasingly showing limitations, including insufficient information fusion and slow computational speed as the number of target structures grows. In contrast, machine learning methods have shown superior performance in identifying binding sites and faster computational efficiency, especially when handling large-scale and complex datasets [12,13,14]. As a result, the continued development of machine learning methods for drug-target binding sites identification is expected to accelerate the drug discovery process [15]. For instance, prediction models of ATP binding sites can not only improve the efficiency of drug screening, but also assist in identifying potential drug targets, designing more accurate targeted drugs, and supporting new drug development.

In modern drug development, computation-based drug screening and wet-lab experiments for drug activity verification are two critical steps, with drug screening playing a critical role in narrowing the scope of wet-lab experiments. Computation-based drug screening process can be divided into three main components: drug-target interaction prediction, drug-target binding affinity prediction, and drug-target binding sites prediction. Research in these areas heavily relies on machine learning techniques. Drug-target interaction prediction models help screen small molecules with activity against specific targets from large molecular databases [16,17,18]. Once potential active small molecules are identified, the next step is to filter out those with weak binding affinities. As a result, machine learning models for predicting drug-target binding affinity have garnered significant attention, including models like MDF-DTA [19], MMDTA [20], and MvGraphDTA [21]. Although drug-target interaction and affinity prediction models can substantially reduce the pool of potential candidates, accurately identifying the specific binding sites of small molecules remains a significant challenge. This is due to the presence of multiple binding pockets on the surface of a target. Consequently, drug-target binding sites prediction models become particularly important. These models can be classified into two categories: classic machine learning methods and deep learning approaches. Classic machine learning methods predict drug-target binding sites by incorporating the shallow features of drugs and targets, such as molecular fingerprints, physicochemical properties of amino acid residues, sequence encodings, etc., into models like random forest [22], support vector machine [23], and XGBoost [24,25]. While these approaches have contributed valuable insights into binding sites identification, their performance is reaching a bottleneck due to their reliance on shallow feature representations of both drugs and targets. In contrast, deep learning methods can leverage not only shallow features but also extract deep-level features that affect binding sites identification. For example, methods [26,27,28] employed 3D convolutional neural networks to extract structural features from the 3D structures of targets. Additionally, models [29,30,31,32] represented the 3D structures of targets as graphs, using graph neural networks to extract structural features and classifying vertices in the graph. Moreover, some methods enhance their prediction capabilities by incorporating deep neural networks and self-attention mechanisms to capture deep-level features of targets [33,34,35]. Despite the advancements made by current drug-target binding sites prediction models, particularly through the integration of diverse data modalities such as physicochemical properties of amino acid residues, target sequences, and target 3D structures, there remains considerable room for performance improvement. Challenges such as insufficient deep-level feature extraction, dataset imbalances, and incomplete model development continue to hinder the full potential of these models in drug-target binding sites prediction.

To further improve the performance of drug-target binding sites identification, we proposed a novel deep learning framework called RGTsite. This framework combined a residual 1D convolutional neural network (1D-CNN) with a residual graph transformer network (GTN) to extract deep-level features from both the sequences and structures of targets. Ultimately, drug-target binding sites were predicted through a fully connected network. Overall, RGTsite made three key contributions as follows.

(1) RGTsite employed a residual 1D-CNN along with the pre-trained model ProtT5 to extract the local and global sequence features from the target. These sequence features were then combined with the physicochemical properties of amino acid residues to construct comprehensive feature representations for each vertex in a target chain graph. Additionally, RGTsite incorporated six-dimensional edge features. These enhancements further strengthened the representational capacity of graph.
(2) Building upon the standard GTN, we introduced a residual technique to aggregate edge features into the vertex features, forming a novel residual GTN. This innovation allowed for a more thorough capture of structural information.
(3) On multiple benchmark datasets, RGTsite exhibited the superior performance compared to the state-of-the-art methods, despite class imbalance, in terms of Matthews Correlation Coefficient (MCC) and F1-score (F1). Furthermore, we conducted interpretability analysis for RGTsite through the specific cases, which further validated the efficiency and effectiveness of RGTsite as a tool for drug-target binding sites identification.

## 2. Materials and Methods

### 2.1. Proposed Model

**Figure 1:**
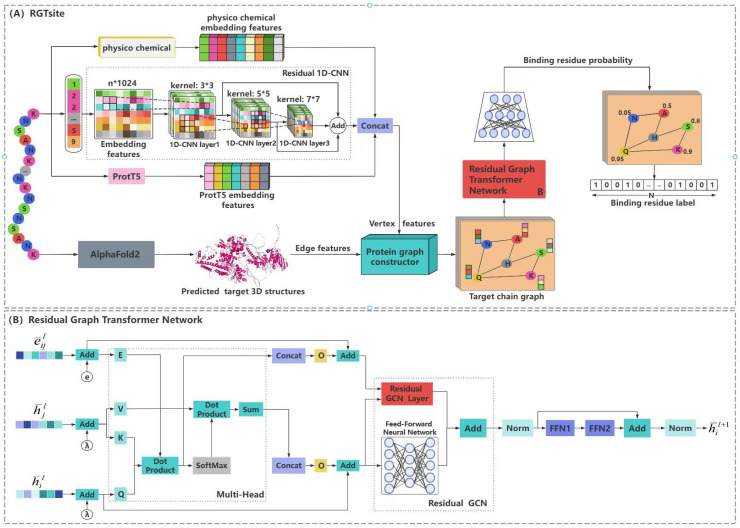
Overview of RGTsite architecture. (**A**) Local and global sequence features extracted by a residual 1D convolutional neural network (1D-CNN) and the pre-trained model ProtT5, respectively, were concatenated with the physicochemical features of amino acid residues to form the vertex features of a target chain graph. After introducing the edge features, a residual graph transformer network (GTN) was employed to obtain the more comprehensive vertex features, which were then processed by a fully connected network to predict whether the vertex represented a binding site. (**B**) A residual GTN was leveraged to integrate edge features into vertex features, thereby enhancing the comprehensiveness of the vertex features and improving the network’s ability to predict binding sites.

In this study, we proposed a drug-target binding sites identification model, RGTsite (Figure 1), which was based on a GTN architecture. The input to RGTsite consisted of three components: target chain graph, vertex features, and edge features. The construction of the target chain graph and edge features are detailed in Section 2.3, while the vertex features were composed of both sequence features and physicochemical features of amino acid residues: (1) A label encoding representation of the target sequence, which was first mapped into a 1024-dimensional feature matrix through a predefined vocabulary and embedding dimension (embedding layer). This feature matrix was then passed through a 3-layer residual 1D-CNN, with padding sizes of 1, 2, and 3 for each layer and convolution kernels of 3 × 3, 5 × 5, and 7 × 7 for the respective layers. This process resulted in a 1024-dimensional representation of local sequence features. (2) After inputting the target sequence into the pre-trained model ProtT5, a 1024-dimensional global sequence feature matrix was obtained for each amino acid residue. (3) Finally, by concatenating the 36-dimensional physicochemical features of amino acid residue with the obtained sequence features, a 2084-dimensional feature matrix was generated and used as the vertex features for the target chain graph.

The target chain graph, vertex features, and edge features were fed into the residual GTN. First, a multi-head attention mechanism (with 8 heads) was employed to extract the vertex and edge features, which were then linearly transformed through a fully connected layer. Using the residual connection method, the original vertex features and edge features were added element-wise to the transformed features, updating both the vertex features and edge features. Next, the updated features were passed into a graph convolutional network with a single hidden layer (256 neurons) to extract the deeper vertex features. Simultaneously, the updated vertex features were input into a fully connected network for linear transformation and then added to the vertex features output by the graph convolutional network. Finally, after undergoing normalization, dropout, feedforward neural network processing, and residual operation, the network output a 256-dimensional vertex feature matrix. This matrix was then fed into a three-layer fully connected network, with sigmoid activation functions applied between layers. The network architecture comprised 64, 16, and 1 neuron in each layer, respectively, to predict drug-target binding sites.

### 2.2. Datasets

In this study, we employed three standard datasets, including PATP-429, PATP-1930, and PATP-NW30 (Table 1). These datasets consisted of target sequences bound to ATP, and these sequences were sourced from the Protein Data Bank (PDB), with variations in data collection years and preprocessing methods. As of 5 November 2016, PATP-429 was constructed, containing 429 sequences. Redundant sequences with greater than 40% sequence consistency were removed using the CD-HIT tool [36]. By 23 June 2023, the NW-Align tool [37] was used to cluster sequences at 40% sequence identity, resulting in the creation of PATP-1930. Additionally, using NW-Align with a 30% sequence identify threshold, PATP-NW30 was generated. As shown in Table 1, it was noted that PATP-1930 was not divided into an independent test set, instead, it directly used the test set of PATP-429. In this process, amino acid residues that bind to ATP were labeled as positive samples, while other residues in the chain served as negative samples. There were significant differences in the positive-to-negative sample ratios in the training and test sets across PATP-429, PATP-1930, and PATP-NW30, all of which were typical imbalanced datasets.

### 2.3. Feature Representation

We employed two ways to represent the sequence features of a target chain. (1) Label encoding: Each target chain generally contained 20 common amino acids, each represented by a number from 1 to 20. Any uncommon amino acids were grouped together and represented by the number 21. (2) Pre-trained model for feature extraction: Given the large number of target sequences, relying solely on extracting deep-level features from a limited set of sequences would not provide comprehensive representations. To address this issue, we leveraged the widely used pre-trained model ProtT5-XL-U50 (ProtT5) [38], which was pre-trained and fine-tuned on BFD (Big Fantastic Database) [39] and UniRef50 [40], covering target sequences of multiple biological species. ProtT5 was applied to extract a 1024-dimensional feature vector for each amino acid residue in a target chain.

First, we utilized AlphaFold2 [41] to predict the 3D structures of targets. There are two key reasons for choosing AlphaFold2: (1) The 3D structures predicted by AlphaFold2 are particularly useful for identifying binding sites on an ATP-target with unknown structures, and it has strong universality. (2) AlphaFold2, developed by DeepMind, can predict 98.5% of human protein structures. In collaboration with EMBL-EBI, DeepMind has constructed the AlphaFold DB database, which contains a large amount of high-precision protein structure prediction data. While there may be cases where the predicted structure of certain proteins (or parts of them) is inaccurate or “degraded”, such occurrences are rare. Next, we treated the $C_{\alpha }$ atoms of amino acid residues in a target chain as vertices of a graph. The Euclidean distance between every pair of vertices was then calculated. If the distance was less than or equal to 8 Å, an edge was considered to exist between them, and the corresponding entry in the adjacency matrix representing the graph was set to 1. Otherwise, the entry was set to 0.

Physicochemical features of amino acid residues were represented by 16 categories, yielding a total of 36 dimensions (Table 2).

Edge features between amino acid residues were characterized by 6 dimensions (Table 2).

### 2.4. Residual Graph Transformer Network

In the target chain graph $G_{T}$, the features of vertex i are represented by $\alpha_{i}\in R^{d_{n}\times 1}$ ($d_{n}$ is the vertex feature dimension), and the edge features between vertices i and j are represented by $\beta_{ij}\in R^{d_{e}\times 1}$ ($d_{e}$ is the edge feature dimension). After linear projection, the implicit representations of the vertex and edge features are $h_{i}^{0}$ (Formula (1)) and ${\bar{e}}_{ij}^{0}$ (Formula (2)), respectively.

(1) $$h_{i}^{0}=A_{0}\alpha_{i}+a_{0}$$

(2) $${\bar{e}}_{ij}^{0}=B_{0}\beta_{ij}+b_{0}$$

Among them, $A_{0}\in R^{d\times d_{n}}$ and $B_{0}\in R^{d\times d_{e}}$ are learnable parameters, while $a_{0},\;b_{0}\in R^{d}$ are biases (d is the dimension of the feature matrix). To incorporate positional encoding into the vertex features, we use the Laplacian vector as the positional encoding in the graph transformer network. The Laplacian feature vector ∆ of each graph is pre-calculated through the factorization of the graph’s Laplacian matrix (Formulas (3)–(5)).

(3) $${\bar{\lambda }}_{i}^{0}=C_{0}\lambda_{i}+c_{0}$$

(4) $${\bar{h}}_{i}^{0}=h_{i}^{0}+{\bar{\lambda }}_{i}^{0}$$

(5) $$\Delta =I-D^{-\frac{1}{2}}AD^{-\frac{1}{2}}=U^{T}FU$$

In Formulas (3) and (4), k minimum non-trivial feature vectors of i are used to form its corresponding positional encoding representation $\lambda_{i}$ ($\lambda_{i}\in R^{k\times 1}$). Here, $C_{0}\in R^{d_{n}\times k}$ is a learnable parameter, $c_{0}\in R^{d_{n}}$ represents the bias, ${\bar{h}}_{i}^{0}$ and ${\bar{\lambda }}_{i}^{0}$ denote the vertex feature matrix and the positional encoding feature matrix, respectively. In Formula (5), I is the identity matrix, A is the adjacency matrix of graph, D is the degree matrix of graph, and F and U represent the eigenvalues and eigenvectors, respectively. Graph transformer updates the vertex and edge features primarily through the multi-head attention mechanism. The update process for vertex and edge features at the $\left(l+1\right)$-th layer is shown in Formulas (6)–(8).

(6) $$w_{ij}^{k,l}={softmax}_{j}\left(\left(\frac{Q^{k,l}{\bar{h}}_{i}^{l}\cdot K^{k,l}{\bar{h}}_{j}^{l}}{\sqrt{d_{k}}}\right)\right)\cdot E^{k,l}{\bar{e}}_{ij}^{l}$$

(7) $${\tilde{h}}_{i}^{l+1}={\bar{h}}_{i}^{l}+O_{h}^{l}\left({Concate}_{k=1}^{gt}\left(\sum_{j\epsilon N_{i}}w_{ij}^{k,l}V^{k,l}{\bar{h}}_{i}^{l}\right)\right)$$

(8) $${\tilde{e}}_{ij}^{l+1}={\bar{e}}_{ij}^{l}+O_{e}^{l}\left({Concat}_{k=1}^{gt}\left(w_{ij}^{k,l}\right)\right)$$

$Q^{k,l}$, $K^{k,l}$, $V^{k,l}$, $E^{k,l}\in R^{d_{k}\times d}$, $O_{h}^{l}$, and $O_{e}^{l}\in R^{d\times d}$ are learnable parameters, where $k\in \left[1,\;gt\right]$ represents the number of heads and $d_{k}$ indicates the dimension of each head. $w_{ij}^{k,l}$ signifies the self-attention weight matrix incorporating the edge features. The resulting edge feature matrix is used as the edge weight matrix $A_{ew}^{T}\in R^{N_{x}^{T}\times N_{x}^{T}}$, from which the adjacency matrix ${\tilde{A}}_{x_{ew}}^{T}$ of the Laplacian edge weighted vertex x is derived (Formula (9)).

(9) $${\tilde{A}}_{x_{ew}}^{T}={\left(D_{x}^{T}\right)}^{-\frac{1}{2}}\left(\left(A_{x}^{T}+I_{N_{x}^{T}}^{T}\right)⨂\left(A_{ew}^{T}+I_{N_{x}^{T}}^{T}\right)\right){\left(D_{x}^{T}\right)}^{-\frac{1}{2}}$$

$D_{x}^{T}$ is the diagonal matrix of $A_{x}^{T}+I_{N_{x}^{T}}^{T}$, where $A_{x}^{T}$ and $I_{N_{x}^{T}}^{T}$ represent the adjacency matrix and identity matrix, respectively. $N_{x}^{T}$ denotes the dimension of the feature matrix, ew represents the edge weight, and T indicates the target. By using ${\tilde{A}}_{x_{ew}}^{T}$ and the residual vertex feature ${H_{x}^{T}}^{\left(l\right)}$ from the l-th layer, the residual vertex feature ${\tilde{h}}_{i}^{l+1}$ of the $\left(l+1\right)$-th layer is obtained (Formula (10)).

(10) $${\tilde{h}}_{i}^{l+1}=\sigma \left({\tilde{A}}_{x_{ew}}^{T}\left({H_{x}^{T}}^{\left(l\right)}\right)W_{x}^{T}\right)+MLP\left({H_{x}^{T}}^{\left(l\right)}\right)$$

$W_{x}^{T}$ represents the learnable parameter matrix. ${\tilde{h}}_{i}^{l+1}$ is fed into the feedforward neural network, where the vertex features $h_{i}^{l+1}$ are obtained through normalization and activation function operations (Formula (11)).

(11) $$h_{i}^{l+1}={\tilde{h}}_{i}^{l+1}+W_{{\tilde{h}}_{1}}^{l}\left(ReLU\left(W_{{\tilde{h}}_{2}}^{l}Norm\left({\tilde{h}}_{i}^{l+1}\right)\right)\right)$$

$W_{{\tilde{h}}_{1}}^{l}$ and $W_{{\tilde{h}}_{2}}^{l}$ are the learnable parameter matrices.

### 2.5. Model Training

In this study, to prevent overfitting, we adopted an early-stopping mechanism during RGTsite training. The early-stopping strategy was as follows: 10% of target chains from the training set were randomly selected as the validation set. If the validation loss did not decrease for 10 consecutive epochs during training, the process was terminated. The optimizer, activation function, and asymmetric loss function used in RGTsite were Adam, ReLU, and Asymmetric Loss (ASL) [42] (Formula (12)), respectively. The learning rate, weight decay, numerical stability parameters, and momentum coefficient were set to 0.0001, 5 × 10^−5^, 1 × 10^−7^, and (0.9, 0.999), respectively. The batch size and dropout rate were set to 1 and 0.3, respectively.

(12) $$ASL=\left\{\begin{array}{c} L_{+}={\left(1-p\right)}^{\gamma }+\log\left(p\right)\\ {\;L}_{-}={\left(p_{m}\right)}^{\gamma }-\log\left(1-p_{m}\right) \end{array}\right.$$

Among them, p represents the model’s prediction probability, γ is the focusing parameter, $p_{m}$ is the offset-adjusted probability, and $L_{+}$ and $L_{-}$ denote the positive and negative loss components, respectively.

### 2.6. Evaluation Metrics

To evaluate the model’s performance, we used sensitivity (Sen), specificity (Spe), accuracy (Acc), precision (Pre), Matthews Correlation Coefficient (MCC), and F1-score (F1).

$$Sen=\frac{TP}{TP+FN}\times 100$$

$$Spe=\frac{TN}{TN+FP}\times 100$$

$$Acc=\frac{TP+TN}{TP+TN+FP+FN}\times 100$$

$$Pre=\frac{TP}{TP+FP}\times 100$$

$$MCC=\frac{TP\cdot TN-FP\cdot FN}{\sqrt{\left(TP+FN)\cdot (TP+FP)\cdot (TN+FN)\cdot (TN+FP\right)}}$$

$$F_{1}=\frac{2\cdot Sen\cdot Pre}{Sen+Pre}\times 100$$

Among them, TP, FN, TN, and FP represent true positives, false negatives, true negatives, and false positives, respectively. Both F1 and MCC provide a more accurate assessment of the model’s performance on imbalanced datasets.

## 3. Results

### 3.1. Performance Comparison Between RGTsite and State-of-the-Art Methods

In this section, we evaluated the performance of RGTsite by comparing it with existing state-of-the-art methods for predicting protein-ATP binding sites, both sequence-based and structure-based.

Sequence-based methods included NsitePred [43], TargetS [44], ATPseq [45], DELIA (seq) [46], DeepATPseq [47], and E2EATP (388, 1930) [12]. Among these, DELIA (seq) referred to DELIA trained only on sequence-based features, and E2EATP (388, 1930) represented the versions of E2EATP trained on PATP-388 and PATP-1930, respectively. We compared the performance of these methods against RGTsite on the independent test set PATP-41. To provide a comprehensive evaluation for RGTsite, we also trained RGTsite (388) and RGTsite (1930) on PATP-388 and PATP-1930, respectively. Experimental results (Table 3) showed that RGTsite (388) outperformed NsitePred, TargetS, ATPseq, DELIA (seq), DeepATPseq, and E2EATP (388) across all evaluation metrics. Notably, RGTsite (388) exhibited improvements in key metrics, achieving F1 (70.16%) and MCC (0.699), surpassing the best-performing methods by 4.14% and 6.72%, respectively, compared to their optimal values (66.02%, 0.655). RGTsite (1930) also outperformed all comparison methods, with the exception of a slight decrease in Spe by 0.18% and Pre by 0.56% compared to ATPseq. In particular, RGTsite (1930) improved F1 (68.21%) and MCC (0.668) by 3.1% and 5.24%, respectively. The comparison results indicated that incorporating 3D structural information of the target enhanced the performance of drug-target binding sites prediction, when compared to sequence-based methods alone.

Structure-based methods included COACH (ITA, AF2) [48], ATPbind (ITA, AF2) [45], and DELIA (ITA, AF2), where ITA and AF2 indicated that the target 3D structures required for these methods were derived from the structure prediction tools I-TASSER [49] and AlphaFold2 [41], respectively. The independent test set used was PATP-41. In the comparative experiment with structure-based methods, all target 3D structures for RGTsite were sourced from the predicted results of AlphaFold2, without using experimentally determined 3D structures. This approach was also designed to provide services for targets lacking experimental 3D structures. Experimental results (Table 4) revealed that RGTsite (388) outperformed existing structure-based state-of-the-art methods across all evaluation metrices, except for Sen. RGTsite (388) excelled in F1 and MCC metrices, showing improvements of 3.1% and 5.27%, respectively, compared to the best-performing method, ATPbind (AF2). Overall, RGTsite’s performance surpassed that of these methods. Specifically, RGTsite (388) achieved an F1 score of 70.16%, and an MCC value of 0.699. RGTsite (1930) showed a 2.67% decrease in Sen compared to COACH (AF2), and a 0.1% in Spe and a 0.05% decrease in Pre, compared to ATPbind (AF2), but outperformed all state-of-the-art methods across other evaluation metrics, particularly in F1 (71.31%) and MCC (0.703), with improvements of 4.25% and 5.87%, respectively, over the best-performing method ATPbind (AF2). Comparisons with state-of-the-art structure-based methods had demonstrated that RGTsite was highly effective in extracting features from vertices (amino acid residues) and in making more accurate predictions regarding binding sites. Additionally, the advantage of applying residue GTN in RGTsite had been confirmed.

To further comprehensively evaluate the performance of RGTsite, we compared it with a series of state-of-the-art methods using the independent test set PATP-NW30-103. Both E2EATP (NW30) and RGTsite (NW30) were trained on PATP-NW30-1861, while the experimental results of other methods were cited from E2EATP. As shown in the results (Table 5), RGTsite (NW30) achieved the highest F1 (60.02%) and MCC (0.595) among all methods, with improvements of 2.63% and 5.12%, respectively, compared to the top-performing method. In addition, RGTsite’s performance across the remaining evaluation metrics also reached the levels comparable to the best values. Overall, RGTsite’s excellent performance on PATP-NW30 demonstrated that the residual GTN-based approach effectively extracted the key features influencing vertex (amino acid residue) classification across different datasets. This highlighted RGTsite’s strong vertex classification capabilities, making it suitable for analysis tasks on complex datasets.

### 3.2. Impact of Three Types of Features That Make up the Vertex Features on the Model’s Performance

In this study, we integrated the global sequence features output by ProtT5, the local sequence features extracted by a residual 1D-CNN, and the physicochemical features of amino acid residues as the vertex features within the residual GTN. To assess the effectiveness of these three types of features, we combined them and constructed six different variant models (Variant 1–Variant 6). Using PATP-1930 as the training set and PATP-41 as the independent test set, we conducted comparative experiments between RGTsite and six variant models. Experimental results (Table 6) revealed that while RGTsite, which combined all three types of features, achieved comparable or slightly inferior performance compared to the variant models in terms of Sen, Spe, Acc, and Pre, it outperformed all variant models in F1 and MCC. Specifically, RGTsite achieved F1 (71.31%) and MCC (0.703), which were 1.24% and 0.57% higher, respectively, than the corresponding best results obtained by the variant models. These findings clearly showed that combining the three types of features had a positive impact on enhancing the overall performance of the model.

### 3.3. Residual 1D-CNN Helps Improve the Model’s Performance

Here, residual 1D-CNN was employed to extract the local sequence features. However, there were various models available for extracting the sequence features of target, such as 1D-CNN, recurrent neural networks (RNN), and transformer. Given that transformer was already incorporated in the residual GTN, we aimed to avoid redundant feature extraction and therefore only conducted comparative experiments with RNN and 1D-CNN to validate the effectiveness of residual 1D-CNN. It was worth noting that we adapted an improved three-layer residual 1D-CNN architecture which incorporated two residual connections built upon 1D-CNN. Under the experimental conditions of using PATP-1930 and PATP-41 as the training and test sets, respectively, experimental results (Table 7) showed that RNN outperformed 1D-CNN in terms of Sen, but 1D-CNN showed superior performance across the remaining evaluation metrics. This outcome may be attributed to the redundancy between the global sequence features extracted by RNN and those output by ProtT5, while the local sequence features extracted by 1D-CNN complement the global features from ProtT5 more effectively. Although 1D-CNN demonstrated better performance compared to RNN, the residual 1D-CNN introduced in RGTsite outperformed 1D-CNN in two key evaluation metrics: F1 and MCC. This suggested that the local sequence features extracted by the residual 1D-CNN played a significant role in improving the performance of drug-target binding sites prediction model.

### 3.4. Residual Graph Transformer Network Is Beneficial for Identifying Binding Sites

Residual GTN served as the core architecture in RGTsite, capturing the vertex feature information by inputting a target chain graph, vertex features, and edge features. To verify the effectiveness of this core architecture, we conducted comparative experiments using widely adopted graph neural network models, including graph convolutional network (GCN), graph attention network (GAT), graph isomorphism network (GIN), GraphSAGE, and GTN, on the training set PATP-1930 and test set PATP-41. Experimental results (Table 8) revealed that GCN and GAT performed relatively poorly, while GIN, GraphSAGE, and GTN all achieved an F1 above 60% and an MCC above 0.6, with the other evaluation metrics being comparable across these models. Notably, GTN outperformed the others in both F1 and MCC, achieving scores of 68.97% and 0.692, respectively. Although GTN exhibited strong performance, it did not incorporate edge features. In contrast, RGTsite, which leveraged residual GTN as its core architecture, introduced the edge features through residual connections, thereby enriching the vertex-feature extraction process. Experimental results confirmed that RGTsite not only performed on par with or better than GTN in four evaluation metrics: Sen, Spe, Acc, and Pre, but also surpassed GTN in F1 and MCC. Specifically, RGTsite showed improvements of 2.34% in F1 and 1.59% in MCC.

### 3.5. Case Study

**Figure 2:**
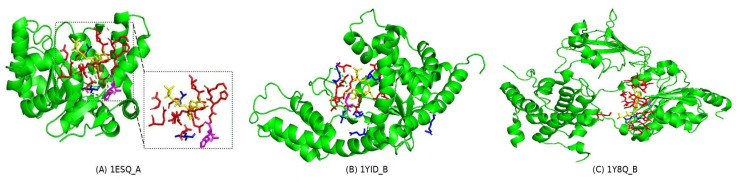
Visual results of binding sites identified by RGTsite, with yellow representing ATP, and green, red, blue, and purple corresponding to amino acid residues classified as TN (True Negative), TP (True Positive), FP (False Positive), and FN (False Negative), respectively. The identification results for TN, TP, FP, and FN amino acid residues on target chains 1ESQ_A, 1YID_B, and 1Y8Q_B were presented in (**A**–**C**), respectively.

To evaluate the performance of RGTsite in practical applications, we selected 20 and 149 target chains from the dataset used by the state-of-the-art method ATP-Deep [50] and constructed two external validation datasets PATP_20 (Table 9) and PATP_149, respectively. All chains in these datasets were excluded from any benchmark sets used in this study. For each chain, PATP_20 contained the following information: target_chain_name, the number of non-binding sites (True_label_0), and the number of binding sites (True_label_1). We processed these chains one by one through RGTsite and recorded the predicted number of non-binding sites (Predicted_label_0), the predicted number of binding sites (Predicted_label_1), the F1, and the MCC. Experimental results (Table 9) showed that RGTsite achieved an F1 of over 80% for 80% of the target chains, and an MCC of over 0.8 for 75% of the target chains. Moreover, the mean F1 and MCC values of all target chains were 86.72% and 0.867, respectively, both of which outperformed RGTsite’s performance on its benchmark test sets. Furthermore, we visualized the binding sites identification by RGTsite on three target chains (Figure 2). By examining the colors of the identified amino acid residues (green: True Negative (TN), red: True Positive (TP), blue: False Positive (FP), and purple: False Negative (FN)) around ATP (yellow), it was intuitively clear that the true binding sites (red) of ATP had been accurately identified. RGTsite’s outstanding performance on PATP_20 demonstrated its reliability as a drug-target binding site prediction tool in practical applications.

We also assessed the practical application capability of RGTsite using PATP_149. Experimental results (Table 10) showed that RGTsite performed well across all evaluation metrics. RGTsite’s outstanding performance on PATP_20 and PATP_149 demonstrated its reliability as a drug-target binding sites prediction tool in practical applications.

Additionally, to further confirm that RGTsite can predict the binding sites of small molecules beyond ATP, we trained RGTsite on the Pro_Train_335 dataset from the advanced method DeepProSite and assessed its performance with the Pro_Test_60 and Pro_Test_315 test sets. Experimental results (Table 11) showed that RGTsite can predict binding sites for small molecules other than ATP, while also exhibiting good performance.

### 3.6. Interpretability Analysis of RGTsite

**Figure 3:**
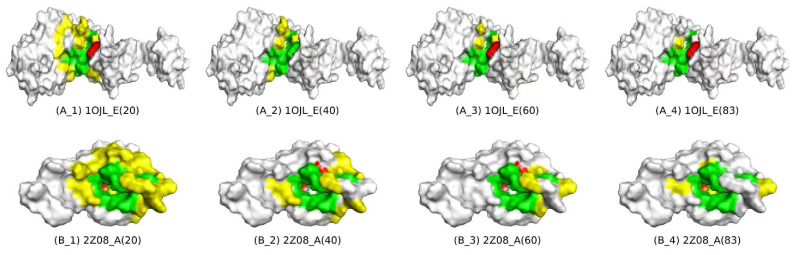
Visualization of amino acid residues identified by RGTsite in the TN (True Negative), TP (True Positive), FP (False Positive), and FN (False Negative) categories at different training epochs. Among them, gray-white, green, yellow, and red represented amino acid residues classified as TN, TP, FP, and FN, respectively. The upper row (**A_1**–**A_4**) and lower row (**B_1**–**B_4**) presented the identification results of RGTsite for the amino acid residues in these categories for the target chains 1OJL_E and 2Z08_A after 20, 40, 60, and 83 training epochs, respectively.

To explain the effectiveness of RGTsite, we saved models trained on PATP-1930 at epochs 20, 40, 60, and 83. We then randomly selected two target chains 1OJL_E and 2Z08_A from PATP_20 and ran these models on them. For each model, we counted the number of amino acid residues classified as TN, FN, FP, and TP, as well as the corresponding F1 and MCC values (Table 12). Statistical results showed that with an increasing number of training epochs, RGTsite gradually reduced the misidentification of FP amino acid residues and improved the accuracy of true-binding sites identification. In addition to the statistical data, we visualized the identification results of RGTsite for the target chains after training at epochs 20, 40, 60, and 83 (Figure 3). Among them, gray-white, green, yellow, and red represented amino acid residues classified as TN, TP, FP, and FN, respectively. From the visualization results, it was evident that after 20 epochs of training, RGTsite had mostly identified the binding sites of TP class but still misclassified some non-binding sites as binding sites. As the training epochs increased, the misidentified FP amino acid residues (yellow) gradually decreased, and both the F1 and MCC values improved, ultimately resulting in the accurate identification of binding sites for 1OJL-E and 2Z08-A. Through a comprehensive analysis of both the statistical data and visualization results across different training epochs, it was clear that RGTsite exhibited high effectiveness in predicting drug-target binding sites.

## 4. Conclusions

RGTsite was a deep learning-based tool for identifying drug-target binding sites, employing a residual 1D-CNN, the pre-trained model ProtT5, and a residual GTN to extract sequence and structural features from targets. Experimental results on multiple benchmark datasets showed that RGTsite outperformed existing state-of-the-art methods in two key evaluation metrics: F1 and MCC. Practical application results and interpretability analysis confirmed that RGTsite was an effective and reliable tool for drug-target binding sites identification. Additionally, RGTsite is also effective for identifying binding sites of small molecules beyond ATP.

In the future, we plan to further improve the performance of drug-target binding sites identification in the following five aspects: (1) After decomposing the target into target chains, all amino acid residues in some chains do not participate in binding sites formation. These chains can be preprocessed to reduce the number of negative samples, thereby alleviating the class imbalance in the dataset; (2) RGTsite is currently a single task model focused solely on predicting drug-target binding sites. We will explore to introduce a drug-target binding affinity prediction task, fusing the deep features extracted from both tasks to create a mutually reinforcing multi-task learning model; (3) While RGTsite has achieved performance improvement in drug-target binding sites identification through the residual GTN architecture, its F1 and MCC are just exceeded 70%, still falling short of user expectations. Continued advancements in deep learning techniques are needed to design innovative drug-target binding sites identification models that can further enhance the prediction performance; (4) We will incorporate the effects of conformational changes on binding site variations to broaden the application scope of RGTsite; (5) Exploring the similarities between different binding pockets to enhance RGTsite’s ability to learn key implicit features.

## Figures and Tables

**Table 1:** Statistical analysis of benchmark datasets.

Datasets	Sequences	Training Set	Test Set	Samples in Training Set			Samples in Test Set		
				Positive Samples	Negative Samples	Positive-Negative Ratio	Positive Samples	Negative Samples	Positive-Negative Ratio
PATP-429	429	388	41	5657	142,086	1:25.12	674	14,159	1:21.01
PATP-1930	1930	1930	41	32,695	709,747	1:21.71	674	14,159	1:21.01
PATP-NW30	1964	1861	103	30,550	813,587	1:26.63	1552	42,034	1:28.08

**Table 2:** Physicochemical features of amino acid residues and edge features.

Type	Feature	Description	Dimension
Physicochemical features	Residue type	‘A’, ‘C’, ‘D’, ‘E’, ‘F’, ‘G’, ‘H’, ‘I’, ‘K’, ‘L’, ‘M’, ‘N’, ‘P’, ‘Q’, ‘R’, ‘S’, ‘T’, ‘V’, ‘W’, ‘Y’, ‘X’	21
	Residue molecular weight	molecular weight values	1
	Residue pKa	pKa values	1
	Residue pKb	pKb values	1
	Residue pKx	pKx values	1
	Residue hydrophobicity (pH = 2)	hydrophobicity values with pH = 2	1
	Residue hydrophobicity (pH = 7)	hydrophobicity values with pH = 7	1
	Residue max_distance	max_distance value of all atoms within the residue	1
	Residue min_distance	min_distance value of all atoms within the residue	1
	Residue CA-O distance	distance values	1
	Residue O-N distance	distance values	1
	Residue N-C distance	distance values	1
	Phi angle (φ angle)	0 or Phi values	1
	Psi angle (ψ angle)	0 or Psi values	1
	Omega angle (ω angle)	0 or Omega values	1
	Chi1 angle (χ1 angle)	0 or Chi1 values	1
Edge features	Edge connection	0 or 1	1
	Distance	distance between the $C_{\alpha }$ atoms	1
	Distance	distance between the $C_{\alpha }$ Center Position	1
	Minimum residue distance	min_distance between all residues	1
	Maximum residue distance	max_distances between all residues	1
	Similarity	calculating the cosine similarity of the angle between the vectors of two residues	1

**Table 3:** Performance comparison between RGTsite and sequence-based state-of-the-art methods.

Model	Sen (%)	Spe (%)	Acc (%)	Pre (%)	F1 (%)	MCC
NsitePred	46.74	97.70	95.39	49.22	47.95	0.456
TargetS	51.63	98.89	96.47	68.91	59.03	0.580
ATPseq	54.45	99.27	97.24	78.09	64.16	0.639
DELIA (seq)	55.00	N/A	N/A	71.50	62.17	0.612
DeepATPseq	57.24	99.22	97.32	77.71	65.92	0.655
E2EATP (388)	60.39	98.93	97.18	72.81	66.02	0.649
E2EATP (1930)	65.58	98.73	97.22	71.06	68.21	0.668
RGTsite (388)	60.53	99.43	97.66	83.44	70.16	0.699
RGTsite (1930)	66.02	99.09	97.59	77.53	71.31	0.703

**Table 4:** Performance comparison between RGTsite and structure-based state-of-the-art methods.

Model	Sen (%)	Spe (%)	Acc (%)	Pre (%)	F1 (%)	MCC
COACH (ITA)	58.16	98.59	96.76	66.33	61.98	0.604
COACH (AF2)	68.69	98.05	96.72	62.65	65.53	0.639
ATPbind (ITA)	62.31	98.85	97.19	72.04	66.82	0.656
ATPbind (AF2)	59.05	99.19	97.36	77.58	67.06	0.664
DELIA (ITA)	62.17	98.67	97.01	69.03	65.42	0.640
DELIA (AF2)	62.46	98.82	97.17	71.60	66.72	0.654
RGTsite (388)	60.53	99.43	97.66	83.44	70.16	0.699
RGTsite (1930)	66.02	99.09	97.59	77.53	71.31	0.703

**Table 5:** Performance comparison between RGTsite and state-of-the-art methods based on PATP-NW30-103.

Model	Sen (%)	Spe (%)	Acc (%)	Pre (%)	F1 (%)	MCC
DeepATPseq	35.76	99.58	97.31	75.82	48.60	0.510
ATPseq	33.38	99.11	96.77	58.13	42.41	0.425
COACH (AF2)	55.67	98.16	96.65	52.87	54.24	0.525
ATPbind (AF2)	43.23	99.44	97.43	73.89	54.55	0.553
DELIA (AF2)	44.59	98.89	96.96	59.81	51.09	0.501
E2EATP (NW30)	50.64	99.05	97.32	66.22	57.39	0.566
RGTsite (NW30)	52.00	99.21	97.53	70.98	60.02	0.595

**Table 6:** Performance comparison of different combinations of three types of features that make up vertex features.

Model	A	B	C	Sen (%)	Spe (%)	Acc (%)	Pre (%)	F1 (%)	MCC
Variant 1	√	×	×	82.49	92.14	91.70	33.31	47.46	0.492
Variant 2	×	√	×	64.09	99.10	97.51	77.28	70.07	0.691
Variant 3	×	×	√	83.38	91.94	91.55	33.00	47.29	0.492
Variant 4	√	√	×	56.23	99.70	97.73	90.02	69.22	0.692
Variant 5	√	×	√	81.90	96.16	95.51	50.36	62.37	0.621
Variant 6	×	√	√	58.31	99.57	97.69	86.56	69.68	0.699
RGTsite	√	√	√	66.02	99.09	97.59	77.53	71.31	0.703

A: The global sequence features output by ProtT5. B: The local sequence features extracted by a residual 1D-CNN. C: The physicochemical features of amino acid residues.

**Table 7:** Performance comparison of commonly sequence feature extraction models of target.

Model	Sen (%)	Spe (%)	Acc (%)	Pre (%)	F1 (%)	MCC
RNN	61.87	98.32	96.66	63.66	62.75	0.610
1D-CNN	54.01	99.81	97.73	93.09	68.36	0.700
RGTsite	66.02	99.09	97.59	77.53	71.31	0.703

**Table 8:** Performance comparison of different graph neural network models.

Model	Sen (%)	Spe (%)	Acc (%)	Pre (%)	F1 (%)	MCC
GCN	53.12	98.85	96.77	68.71	59.92	0.588
GAT	1.00	0.00	4.54	4.54	8.69	N/A
GIN	56.53	98.73	96.81	67.91	61.70	0.603
GraphSAGE	60.24	99.23	97.46	78.83	68.29	0.677
GTN	57.86	99.53	97.63	85.34	68.97	0.692
RGTsite	66.02	99.09	97.59	77.53	71.31	0.703

**Table 9:** Statistical results of RGTsite based on PATP_20.

Target_Chain_Name	True_Label_0	Predicted_Label_0	True_Label_1	Predicted_Label_1	F1 (%)	MCC
2BEK_A	238	238	19	19	100	1
1OJL_E	289	289	15	15	93.33	0.93
1Y56_A	472	465	21	28	85.71	0.86
1N5I_A	210	207	4	7	72.73	0.751
3HAV_A	286	287	13	12	96	0.959
1Y8Q_B	621	620	19	20	92.31	0.921
1A49_A	516	514	14	16	93.33	0.934
2HS0_A	570	563	33	40	82.19	0.815
1ESQ_A	269	267	15	17	87.5	0.869
1YID_B	340	333	11	18	68.97	0.7
1NSF_A	253	253	20	20	80	0.784
1QHG_A	714	705	10	19	68.97	0.721
1H4Q_B	462	461	15	16	77.42	0.767
1KVK_A	379	375	16	20	83.33	0.831
1JJV_A	194	194	12	12	91.67	0.912
1MJH_A	142	141	20	21	92.68	0.917
3K5H_A	383	383	20	20	95	0.947
2Z08_A	118	114	19	23	85.71	0.837
3MEY_A	328	329	18	17	97.14	0.97
3C5E_A	551	547	19	23	90.48	0.906

**Table 10:** Practical application capability of RGTsite under PATP_149.

Model	Sen (%)	Spe (%)	Acc (%)	Pre (%)	F1 (%)	MCC
RGTsite	87.15	98.79	98.34	73.96	80.01	0.794

**Table 11:** Performance comparison of RGTsite and DeepProSite.

Model	Dataset	Acc (%)	Pre (%)	F1 (%)	MCC	AUC
DeepProSite	Pro_Test_60	84.2%	50.1%	47.0%	0.379	0.813
RGTsite		84.2%	49.8%	46.1%	0.370	0.710
DeepProSite	Pro_Test_315	80.4%	37.8%	45.7%	0.355	0.805
RGTsite		86.9%	56.6%	43.5%	0.378	0.722

**Table 12:** Performance comparison of RGTsite under different training epochs.

Target_Chain_Name	Epochs	TN	FN	FP	TP	F1 (%)	MCC
1OJL_E	20	262	1	27	14	50	0.533
	40	281	0	8	15	78.95	0.796
	60	285	1	4	14	84.85	0.844
	83	288	1	1	14	93.33	0.93
2Z08_A	20	83	1	35	18	50	0.462
	40	105	2	13	17	69.39	0.656
	60	112	2	6	17	80.95	0.78
	83	113	1	5	18	85.71	0.837

## Data Availability

The dataset, source code, and trained RGTsite model are available at https://github.com/dldxzx/RGTsite (accessed on 17 January 2025).
